# Fluoroquinolone-Resistant *Alcaligenes faecalis* Related to Chronic Suppurative Otitis Media, Angola

**DOI:** 10.3201/eid2310.170268

**Published:** 2017-10

**Authors:** Matuba Filipe, Åke Reimer, Erika Matuschek, Maria Paul, Tuula Pelkonen, Kristian Riesbeck

**Affiliations:** Hospital Josina Machel, Luanda, Angola (M. Filipe);; Slottsstadens Läkarhus, Malmö, Sweden (Å. Reimer);; Faculty of Medicine Lund University, Malmö (Å. Reimer, M. Paul, K. Riesbeck);; EUCAST Development Laboratory, Central Hospital, Växjö, Sweden (E. Matuschek);; Children’s Hospital, Helsinki University Hospital; and Helisinki University, Helsinki, Finland (T. Pelkonen)

**Keywords:** Alcaligenes faecalis, Pseudomonas aeruginosa, Proteus mirabilis, enterococci, MRSA and other staphylococci, child, infection, chronic suppurative otitis media, CSOM, ear, discharge, peritonitis, eye, urinary tract, bird feces, Luanda, Angola, bacteria

## Abstract

We found that 20 (10.6%) of 188 patients with chronic suppurative otitis media in Angola were co-colonized with fluoroquinolone-resistant *Alcaligenes faecalis*, commonly found in birds. A likely explanation for our findings was the use of bird feces by residents as a traditional remedy to prevent ear secretions caused by primary ear infection.

Chronic suppurative otitis media (CSOM) is a common condition in developing countries and in original populations such as the Inuit (*1*). Mainly, young children are reported to have CSOM; 30%–40% of the population are affected in some geographic areas (*2*). CSOM is associated with hearing loss caused by perforation of the tympanic membrane, which often leads to chronic infection of the middle ear. Overcrowding, poor hygiene, and low nutrition status, in addition to absence of modern healthcare systems, are contributing factors for CSOM (*2*). CSOM is caused by a polymicrobial infection including gram-negative species, dominated by *Pseudomonas aeruginosa* and *Proteus mirabilis*, in addition to gram-positive bacteria such as enterococci and staphylococci (*3*).

During studies of ear disharge caused by otitis media, we detected the gram-negative bacillus *Alcaligenes faecalis* in addition to the commonly isolated bacterial species (*3*). *A. faecalis* may reside in the human microbiome of the gastrointestinal tract but only occasionally causes disease. Most cases occur in immunocompromised hosts, but in rare instances, *A. faecalis* infection has been described in patients who had acute otitis media, peritonitis, and eye or urinary tract infections (*4**–**6*). *A. faecalis* is frequently found in bird fecal specimens and can also cause opportunistic infections in these animals (*7*).

We examined specimens from 188 patients who had ear discharge related to otitis media who attended an outpatient ear, nose, and throat clinic in Luanda, Angola, during January–December 2016. This study was approved by the Angolan Medical Council, the director of the Josína Machel Hospital, and the Luanda University Medical Faculty. After cleaning the ear canal with 70% ethanol, we collected discharge with a swab. For nasopharyngeal sampling, we inserted a swab into the nostril past the choana and touched the wall of the nasopharynx. We collected ear and nasopharyngeal samples and stored them in skim milk-tryptone-glucose-glycerol (STGG) medium (*8*) at −70°C (Public Health Laboratory, Luanda, Angola) before transport to the Riesbeck Laboratory (Malmö, Sweden). Clinical specimens were cultured and bacteria analyzed (Technical Appendix). We found that 20 (10.6%) patients were colonized by *A. faecalis* (Table). Among patients harboring *A. faecalis*, 14 were male (1–47 years of age) and 6 were female (2–23 years of age). *A. faecalis* was growing in polymicrobial communities, and *P. aeruginosa* was the predominant species in 10 (50%) of the patients. *Proteus mirabilis* was the second most common bacterium (n = 7), followed by *Klebsiella* spp. and gram-positive cocci. Eight of the patients had otalgia, and the duration of otorrhoea was >12 months in 4 of those patients.

**Table:** Co-colonizing bacterial species, clinical characteristics, and demographics of 20 patients colonized with *Alcaligenes faecalis* who had ear discharge related to otitis media, Luanda, Angola, January–December 2016*

Patient no.	Co-colonizing bacterial species	Patient age, y/sex	Otalgia	Otorrhoea type, duration, mo	Neighborhood of residence
1	*Pseudomonas aeruginosa*, *Escherichia coli*, *Proteus* spp., *Enterococcus faecalis, Enterococcus avium*	10/M	No	Unilateral, 1	Coelho, Viana‡
2	*P. aeruginosa, Proteus mirabilis, Citrobacter freundii*	8/F	No	Bilateral, <0.5	Cazenga, Cazenga‡
3	*P. aeruginosa, P. mirabilis, Morganella morganii, Providencia rettgeri, E. faecalis*	20/M	No	Bilateral, >3	Tala-Hady, Cazenga‡
4	*P. mirabilis, C. freundii, E. faecalis*	8/M	No	Unilateral, >0.5	Hoji-ya-Henda, Cazenga‡
5	*P. aeruginosa, Staphylococcus saprophyticus*	5/F	No	Unilateral, >12	Bairro 6, Viana‡
6	*P. mirabilis, Corynebacterium striatum, Citrobacter amalonaticus*	4/M	No	Bilateral, >12	Antonove, Cazenga‡
7	*Klebsiella pneumoniae, C. freundii*	23/F	Yes	Unilateral, >12	Rangel, Luanda
8	*P. aeruginosa, Providencia stuartii, Stenotrophomonas maltophilia*	17/M	Yes	Unilateral, >6	Asa Branca, Cazenga‡
9	*P. aeruginosa, K. pneumoniae, Enterobacter cloacae, Arthrobacter* spp.	20/F	Yes	Unilateral, <0.5	Asa Branca, Cazenga‡
10	*P. aeruginosa, P. mirabilis, K. pneumoniae, M. morganii*	22/M	Yes	Unilateral, >12	Cazenga, Cazenga‡
11	*P. aeruginosa, P. stuartii, Citrobacter koseri*	7/F	No	Unilateral, >12	Sambizanga, Luanda
12	No other species‡	13/M	No	Unilateral, >12	Saurimo, Saurimo§
13	*M. morgani*	5/M	Yes	Unilateral, >12	Pimba, Saurimo§
14	*Arthrobacter* spp.‡	6/M	No	Bilateral, NA‡	Saurimo, Saurimo§
15	*P. aeruginosa, Streptococcus constellatus*	14/M	Yes	Unilateral, >12	Saurimo, Saurimo§
16	*P. mirabilis, Arthrobacter s*pp., *E. faecalis*	1/M	Yes	Unilateral, >3	Viana, Viana†
17	*P. aeruginosa, Klebsiella oxytoca*	2/M	Yes	Unilateral, >3	Sanzala, Viana†
18	*Arthrobacter* spp.‡	ND/M	Yes	Unilateral, >12	Tshinganja 2, Saurimo†
19	*Arthrobacter* spp., *P. stuartii*	47/M	No	Unilateral, >12	Estalagem, Viana†
20	*Enterococcus faecium*	2/F	NA	NA, NA	NA¶

*A. faecalis isolated from external ear discharge except as indicated. ND, no data; NA, not applicable. †Province Luanda.  ‡A. faecalis isolated from nasopharyngeal swab. §Province Lunda Sul. ¶Province Namib.

We tested A. *faecalis* isolates against a series of selected antimicrobial drugs by using broth microdilution (Technical Appendix Table) and found 100% were susceptible to aminoglycoside amikacin, cephalosporins, and colistin. In addition, 90% were susceptible to gentamicin, tobramycin, and piperacillin/tazobactam, and 75% were susceptible to trimethoprim/sulfamethoxazole. By contrast, most isolates were resistant to the fluoroquinolones ciprofloxacin (100%) and levofloxacin (82.6%).

*A. faecalis* has been described in patients with CSOM (*9*), but for us, this observation was initially an enigma. We considered the possibility of a contaminant from the endogenous fecal microbiome. We found, however, that to prevent ear discharge, patients occasionally filled their external auditory canals with dove or pigeon feces. Some patients in the geographic area also used cockroach paste, palm oil, sweet olive oil, sewing machine oil, or breast milk to prevent ear discharge. The origin of *A. faecalis* from birds would be a likely explanation for the appearance of this particular bacterial species among this study cohort.

Topical treatment using antimicrobial drugs in combination with keeping the ear canal clean and dry is the mainstay of therapy for CSOM (*10*). It remains to be confirmed whether *A. faecalis* colonization plays a crucial role for disease progression or merely is a contaminant. However, the microbiological findings in this study should be a note of caution because all *A. faecalis* isolates we obtained were resistant against the most commonly used fluoroquinolone, ciprofloxacin. An alternative strategy would therefore be to consider colistin as topical treatment or supplement with orally administered amoxicillin/clavulanic acid in the treatment of more severe cases. The supply of topical agents in Luanda is unknown; therefore, the optimal treatment of patients colonized with *A. faecalis* should be determined and appropriate supplies obtained.

Technical Appendix Sampling of ear discharge and nasopahryngeal swabs showing fluoroquinolone-resistant *Alcaligenes* *faecalis* related to chronic suppurative otitis media, Angola.
