# Supplementary material for: Fluoroquinolone-Resistant Alcaligenes faecalis Related to Chronic Suppurative Otitis Media, Angola
Source: Emerg Infect Dis. 2017 Oct;23(10):1740–2. doi: 10.3201/eid2310.170268 (PMC5621561; doi:10.3201/eid2310.170268)
Supplement: Technical Appendix — Sampling of ear discharge and nasopahryngeal swabs showing fluoroquinolone-resistant Alcaligenes faecalis related to chronic suppurative otitis media, Angola. [file 17-0268-Techapp-s1.pdf]

Article DOI: https://doi.org/10.3201/eid2310.170268

Fluoroquinolone-Resistant *Alcaligenes faecalis* Related to Chronic Suppurative Otitis Media, Angola

Technical Appendix

Sampling of Ear Discharge and Nasopahryngeal Swabs

Culture conditions

Clinical specimens were cultured on hematin agar, Columbia CNA agar (Oxoid), and finally UriSelect agar (Oxoid) supplemented with vancomycin incubated at 35.5°C in 5% CO2 or in aerobic conditions (UriSelect) for 16–18 h. Species identification was done by Matrix-assisted laser desorption/ionization – time of flight mass spectrometry (MALDI-TOF MS).

Antimicrobial susceptibility testing

MICs for *A. faecalis* were interpreted according to the European Committee on Antimicrobial Susceptibility Testing breakpoints for *Pseudomonas* (*1*) (Technical Appendix Table). Due to better concordance, we included breakpoints for *Enterobacteriaceae* regarding trimetoprim-sulfamethoxazole. Our observation that most *A. faecalis* isolates were susceptible to gentamicin was in contrast to a previous study showing that *A. faecalis* were resistant against this particular aminoglycoside (*2*).

References

1. European Committee on Antimicrobial Susceptibility Testing. Breakpoint tables for interpretation of MICs and zone diameters. Version 7.0, 2017. http://www.eucast.org/fileadmin/src/media/PDFs/EUCAST_files/Breakpoint_tables/v_7.0_Breakpoint_Tables.pdf

2. Bizet C, Tekaia F, Philippon A. In-vitro susceptibility of *Alcaligenes faecalis* compared with those of other Alcaligenes spp. to antimicrobial agents including seven beta-lactams. J Antimicrob Chemother. 1993;32:907–10. PubMed http://dx.doi.org/10.1093/jac/32.6.907

Technical Appendix Table. MIC distributions for *Alcaligenes faecalis* (20 isolates) and interpretation according to tentative ECOFFs, and EUCAST clinical breakpoints for *Pseudomonas*.*†

Antimicrobial agent	MIC (mg/L)											Tentative ECOFFs*		*Pseudomonas* breakpoints†		
	≤0.125	0.25	0.5	1	2	4	8	16	32	64	≥128	WT	NWT	S	I	R
Piperacillin-tazobactam	–	–	–	**14‡**	**3**	**1**	–	1	–	–	1	18	2	19	–	1
Cefepime	–	–	–	–	–	**1**	**16**	3	–	–	–	20	0	17	–	3
Ceftazidime	–	–	–	**1**	**12**	**5**	–	1	1	–	–	18	2	18	–	2
Ciprofloxacin	–	–	**2**	**5**	**5**	**5**	–	3§	–	–	–	17	3	2	–	18
Levofloxacin	–	–	**4**	**8**	**5**	–	–	3§	–	–	–	17	3	12	–	8
Amikacin	–	–	–	–	–	**14**	**6**	–	–	–	–	20	0	20	–	–
Gentamicin	–	–	–	–	**18**	**2**	–	–	–	–	–	20	0	18	–	2
Tobramycin	–	–	–	–	**18**	**1**	1	–	–	–	–	19	1	18	–	2
Colistin	–	–	–	**7**	**13**	–	–	–	–	–	–	20	0	20	–	–
Trimethoprim-sulfamethoxazole	**14**	**1**	–	–	–	2	1	–	2¶	–	–	15	5	15#	2	3

*Tentative epidemiologic cutoff (ECOFF) values are based on the MIC distributions in this table. EUCAST, European Committee on Antimicrobial Susceptibility Testing antimicrobial susceptibility testing; Bold text and WT indicate wild type; NWT, non-wild type; S, susceptible; I, intermediate; R, resistant. Dashes indicate nonsusceptibility.
†EUCAST Clinical Breakpoint Tables v 7.0 (*1*).
‡≤1 mg/L
§≥16 mg/LfMIC
¶≥32 mg/L
#For trimethoprim-sulfamethoxazole, breakpoints for *Enteobacteriacae* were used.
